# Changes of *Cinchona* distribution over the past two centuries in the northern Andes

**DOI:** 10.1098/rsos.230229

**Published:** 2023-04-12

**Authors:** Carlos E. González-Orozco, Esther García Guillén, Nicolás Cuvi

**Affiliations:** ^1^ Corporación Colombiana de Investigación Agropecuaria (AGROSAVIA). Centro de Investigación La Libertad-Km 14 vía Puerto López, Villavicencio, Meta, Colombia; ^2^ Real Jardín Botánico, CSIC, Madrid, España; ^3^ Departamento de Antropología, Historia y Humanidades, Facultad Latinoamericana de Ciencias Sociales (FLACSO), Quito, Ecuador

**Keywords:** historical biogeography, elevational shifts, tropical Andes, Ecuador, Colombia

## Abstract

The *Cinchona* genus is important for humanity due to its ethnobotanical properties, and in particular its ability to prevent and treat malaria. However, there have been historical changes of *Cinchona* distribution in the tropical Andes that remain undocumented. In the late eighteenth and early nineteenth centuries, several explorers recorded *Cinchona* precise localities in present-day Colombia and Ecuador, countries which harbour about half of the species of the genus, including *C. officinalis*. We compare historical and twentieth-century records to evaluate whether elevational ranges, mean elevation and latitude varied between the two periods. A large expansion of 662.5 m in average elevation for *Cinchona* and 792.5 m in elevational range for *C. officinalis* was found. These findings have implications for the conservation of economically important species and help us understand the impacts of the Anthropocene over time.

## 1. Introduction

**Figure 1.**
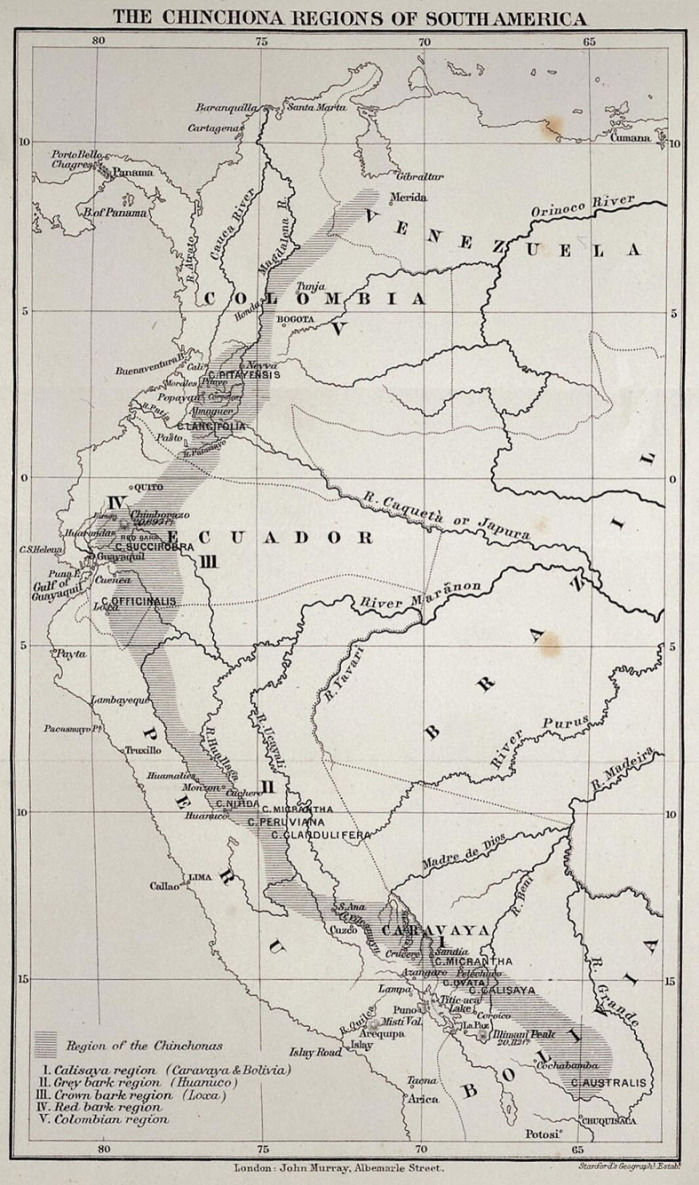
Natural distribution of the genus *Cinchona* in South America [4].

Historic documents can help us understand socio-environmental processes over long periods of time [1,2]. However, the use of old maps and archives to investigate plant taxa in tropical regions, such as *Cinchona* L. (Rubiaceae–Cinchoneae), is limited [3]. The genus is naturally distributed across the Tropical Andes (figure 1) and contains 23 species [5]. The greatest species diversity and endemism are found in southern Ecuador and northern Peru [5,6]. The biogeographical regions of *Cinchona* are composed of three main regions, five subregions and nine provinces [7].

Although there are studies on aspects such as taxonomy and biogeography [8–10], no study has been conducted to explore the historical changes in the distribution of *Cinchona* in present-day Colombia and Ecuador. Our research delves deep into maps, herbaria and archives, to identify changes in distribution, latitude and elevation of the genus and the species *C. officinalis.* We tested whether elevational ranges, mean elevation and latitude have changed over time.

‘Quina’ or ‘cascarilla’ are common names in Spanish for the genus *Cinchona*, and for the close relatives of the genera *Remijia* and *Ladenbergia*. They contain alkaloids that are used to prevent and treat malaria. Each species has different concentrations of alkaloids, which can vary within the same species [11]. They were first used by the Indigenous peoples for medicinal purposes and were later adopted for the same purpose by Europeans in the sixteenth century. Since then, dozens of explorers and merchants have earnestly searched for ‘quinas’ [12]. The first scientific description was made by Charles Marie de la Condamine in 1743, after a short visit to the Loja region, in the south of Ecuador [13]. Joseph de Jussieu was the first botanist to study these plants [14]. Collectors in the late nineteenth century include Clements Markham, Charles Ledger [15] and Hugh Weddell [16].

Since the sixteenth century, *C. officinalis* has been one of the most exploited species in Latin America. As demand grew, the trees and ecosystems of the Loja region showed early signs of over-exploitation [17]. Quina bark gatherers and traders moved north, to the surrounds of the cities of Cuenca and Riobamba, where there were ‘interminable forests’ [18] of *Cinchona*. The eighteenth century witnessed boom and bust periods in several places [19], and 38 years of commercial monopoly of the bark by the Spanish crown took place [20,21]. The Spanish also sent two botanical expeditions to New Granada and Peru to discover useful plants, including new species of *Cinchona* [22–27].

The Royal Botanical Expedition to the Viceroyalty of Peru included Hipólito Ruiz, Joseph Pavón, Joseph Dombey, Juan José Tafalla and Juan Agustín Manzanilla. Tafalla and Manzanilla collected plants in the Loja region, and their works on *Cinchona* were included in the publications of Ruiz and Pavón, and in the *Nueva Quinología* of Pavón [28–30]. Their *Cinchona* collections are held in the Real Jardín Botánico and Kew Gardens and share similar histories [31].

**Figure 2.**
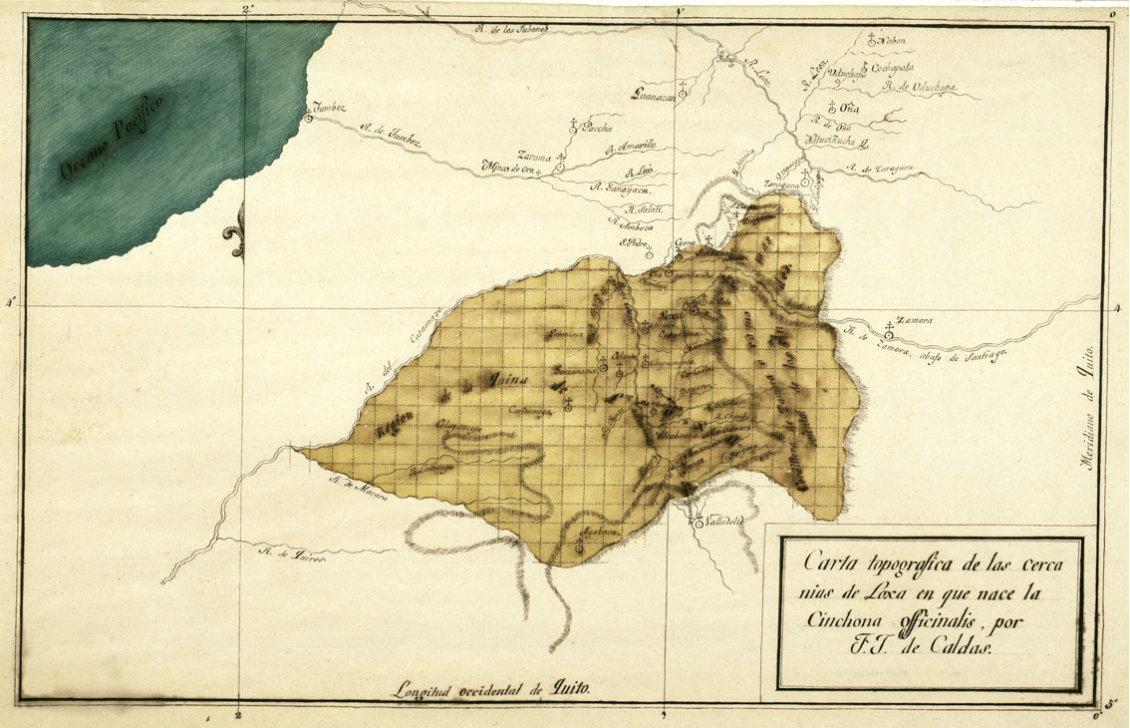
Caldas's gridded map of Loja region, showing the distribution of *C. officinalis,* ‘Topographic map of the *C. officinalis* that grows in the surrounding areas of Loja, by F. J. de Caldas'. AJB, Div. III, M, 528. Image courtesy of Real Jardín Botánico, CSIC.

**Figure 3.**
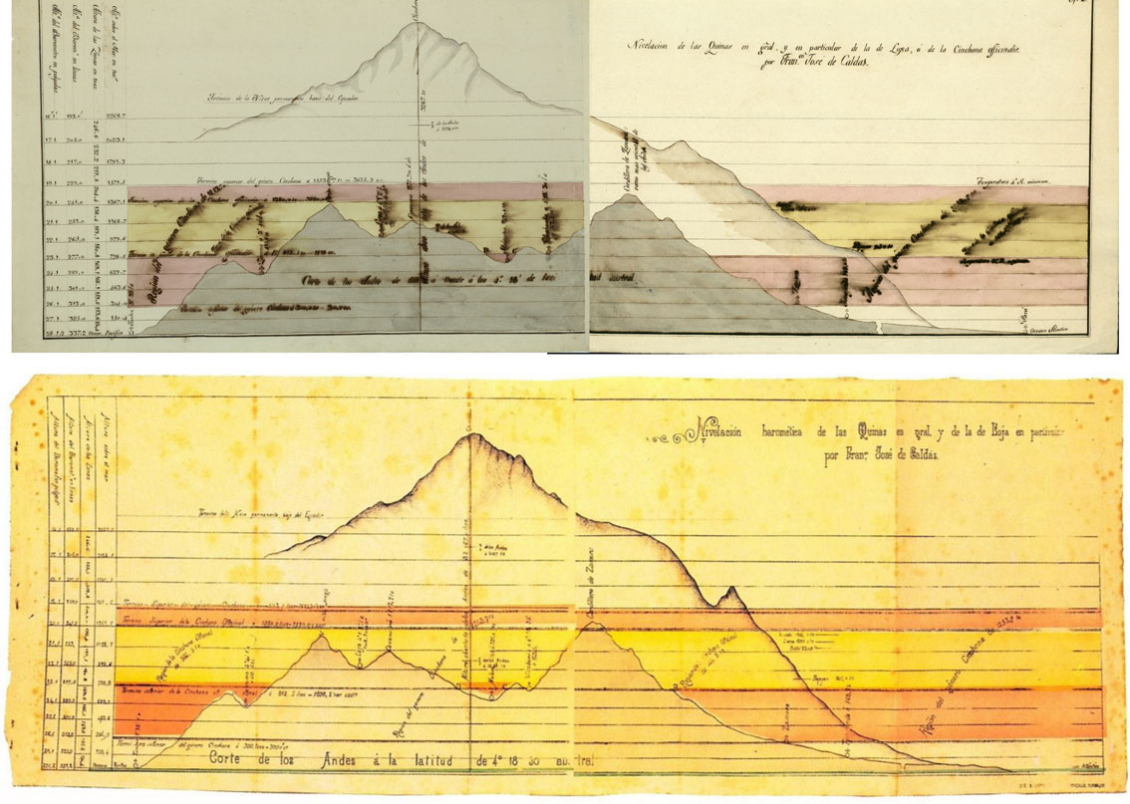
Caldas's Andean profile showing a barometric vertical classification of the elevational limits of the genus *Cinchona* and *C. officinalis* at the latitude of Loja. AJB, Div. III, M, 514 and M, 526. The title of the map, in Spanish, means: ‘Vertical profile of the Quinas in general and particularly the *C. officinalis* of Loja, by F. J. de Caldas'. Images courtesy of Real Jardín Botánico, CSIC. Bottom image is a copy of the original map [43].

In the Viceroyalty of New Granada, the Royal Expedition was led by the Spanish José Celestino Mutis. One of his members, the Negranadean Francisco José de Caldas, carried out extensive collections of *Cinchona*. Many studies have focused on the achievements and controversies around the Royal Botanical Expedition to the New Kingdom of Granada [32–40]. For example, Caldas drew mountain profiles, mapping out vertical zones where wild plants grew, a technique that was first applied to crops by J-L. Giraud Soulavie in France [32–35,38–40]. In the tropics, the technique became known when the Prussian, Alexander von Humboldt, applied it in his *Tableau Physique* [36,37]. Caldas used vertical zones to illustrate the distribution of Andean crops and other plants including the *Cinchona* [43–45]. Using a gridded map, which was innovative for his time, he was able to generate precise registers of latitude and longitude distribution (figure 2) and made a vertical zonation profile of *Cinchona* (figure 3) in the Loja region, and the foothills of the Andes through the Pacific Ocean and to the Amazon. Caldas wrote a report on the general state of the quinas and, in particular, those of Loja [22]. He also prepared a more extensive document, the *Cinchographia*, which was partially published by Carl von Martius [46]. His contribution was essential for the Pars Quarta of the *Historia de los árboles de la quina* [47] (History of the Cinchona Tree).

During this time, French botanist Aimé Bonpland and Humboldt also collected *Cinchona* in Colombia and Ecuador at the same time as Caldas and Mutis. Bonpland kept field notes and provided herbarium collections [48] and included information on the genus in various publications [49–51]. Humboldt also published specific articles on the genus [41,42], although they contained several taxonomic mistakes already pointed out at that time by Caldas [2].

Although some mistakes would have affected the results [52], we consider that the records used are sufficient to explore if there have been elevational and latitudinal changes in *Cinchona* distribution in these regions between the ‘late colonial’ period *(ca* 1800) to the twentieth century (called here the ‘contemporary’ period). Our hypothesis tests the idea that an enlargement of elevational range has occurred.

## 2. Material and methods

### 2.1. Late colonial records

Historical information on *C. officinalis* and the genus *Cinchona* for Ecuador and Colombia was gathered from three groups of historical collectors: Caldas, Humboldt & Bonpland and Tafalla & Manzanilla (table 1; included in electronic supplementary material, appendix, datasets S2,4,5,6). Most of the historical records correspond to present-day Ecuador. Caldas's records in Ecuador were the most precise and extensive; however, we used Humboldt & Bonpland and Tafalla & Manzanilla data to compare and supplement Caldas's data. We did not include records from Venezuela, Peru and Bolivia, nor were any hybrids or infra-specific levels used in our study. In terms of taxonomic status, we rely on Andersson's 1998 *Cinchona* taxonomic classification [5]. We investigated the distributions at the genus level and at the species level *C. officinalis*.

**Table 1.** Summary of the late colonial and contemporary datasets used in our study. (n.a., not applicable).

collectors	dataset no.	source	period	taxon	no sp.	no of records analysed	country
Caldas	S2a	memoirs [24], map of C. officinalis [53] and Andean profiles	late colonial	*Cinchona* sp. and *C. officinalis*	n.a.	54	Colombia and Ecuador
Caldas	S4-5	Martius 1847: 189	late colonial	*Cinchona* sp.	n.a.	7	Colombia
Humboldt & Bonpland	S4-5	publication	late colonial	*Cinchona* sp.	n.a.	11	Colombia and Ecuador
Tafalla & Manzanilla	S4-5	publication	late colonial	*Cinchona* spp.	n.a.	37	Ecuador
Tafalla & Manzanilla	S4-5	publication	late colonial	*C. officinalis*	n.a.	26	Ecuador
various	S3	Gonzalez-Orozco (2021) [12]	contemporary	*Cinchona* sp.	9	668 (not used for analysis)	Colombia
Garmendia	S1	Garmendia (2005) [17]	contemporary	*Cinchona* sp.	12	446	Ecuador
Garmendia, Caldas, Humboldt & Bonpland, Tafalla & Manzanilla	S6	all sources DS4–5	late colonial and contemporary	*Cinchona* spp.	n.a.	95-488	Colombia and Ecuador
Caldas	S2b	memoirs [24]	late colonial and contemporary	*Cinchona* sp.	n.a.	89	Colombia and Ecuador

#### 2.1.1. Caldas

A revision and analysis of several historical sources containing information on the localities of the genus *Cinchona* in Colombia and Ecuador was undertaken. The most important dataset was composed of Caldas's records reported in his *Memoria sobre el estado de las quinas en general y, en particular, sobre las de Loja* (Report on the state of the quinas in general and, in particular, those of Loja) [22]. *Cinchona* localities for Ecuador were also extracted from: the *Pars Quarta* of the Historia de los Arboles de la Quina [23,54]; the topographic map of the *C. officinalis* that grows in the surrounding areas of Loja (figure 2); the vertical profile of Loja regions (figure 3); the profile maps of Caldas's phytogeographical regions in the Andes [55]; and the publication of Carl von Martius [46] that includes the data of the disappeared manuscript of Caldas's Cinchografía.

The original document of the Memoria is in the Archives of the Real Jardín Botánico-CSIC, Madrid. It contains data on localities of *C. officinalis* on a Cartesian map in the Loja region (figure 2) and a vertical profile describing the distribution of quinas (figure 3). Each of the grid cells in the Cartesian map cover 0.05 by 0.05° with an extent from 1°47′ of latitude to 2°30′ longitude across the Andean Mountain range of the Loja region. The profile, on the other hand, is the sheet number 1 of a mosaic that depicts an east to west transect at 4° 18′ of latitude showing the maximum elevation of the Andes with the Chimborazo volcano as a spatial reference in the background. This vertical zonation covers the sea level of the Pacific Ocean across the mountains of the Andes and towards the Amazon forests in the east. The vertical zones are divided into four columns showing the elevation in toises (a French metric of longitude), height of the vertical zones in toises, height of the barometer in lines (metric of distance) and height of the barometer in inches.

Other historical sources of Caldas were distributions of *Cinchona* taken from the profile maps of the Andes [55], charts, field notes, descriptions, drawings and herbarium specimens [56].

Assigning latitude, longitude and elevation to all the reported locations where Caldas observed *Cinchona* and *C. officinalis* in Ecuador and Colombia was key to building Caldas's data collection. Spatial coordinates were assigned to *Cinchona* distributions in the late colonial period using the names of towns and localities reported in the Cartesian map, the vertical profile and the text or tables in the Memoria. Each site was checked in Google Earth to extract the latitude and longitude. We were aware of the location of each site because of our own regional knowledge of the territory, reducing the potential for errors in the spatial accuracy of elevations and geo-positions. The elevation estimates documented by Caldas were converted from toises to metres. A dataset of 54 entries of colonial locations of *Cinchona* in Colombia and Ecuador was obtained (electronic supplementary material, appendix, dataset S2a). Another dataset of 89 entries of colonial and contemporary locations of *Cinchona* in Ecuador was obtained (electronic supplementary material, appendix, dataset S2b). A complementary dataset not available in the Memoria, with seven new historical records of *Cinchona* spp. in Colombia, was also created (included in electronic supplementary material, appendix, dataset S4).

#### 2.1.2. Humboldt and Bonpland

The field notes of Bonpland from his travels together with Humboldt in Ecuador and Colombia were a primary source of information. They are preserved at the Museum National d'Histoire Naturelle, Paris. These records were crossed with the collection numbers and the information in the original labels of his *Cinchona* specimens at the Paris and Berlin Herbariums. Mentions of *Cinchona* and *C. officinalis* (identified by them as *C. condaminea*) in two other publications were also considered by Humboldt and Bonpland [50,51,57].

Bonpland and Humboldt's data varied slightly over time. In the third volume of the Nova Genera Plantarum, the seven species of the genus, collected in the north of Bracamoros, Ecuador, were found at a range between 300 and 1600 hex (approx. 584.7–3118.4 m), and the *C. condaminea*, was found between 900 and 1200 hexapeds (French equivalent of the toesa, approx. 1754.1–2338.8 m) [51]. This is consistent with the findings in Humbold's Essay on the Geography of Plants, which states ‘we have not encountered any fever tree at less than 700 m (359 toesas) above sea level and no one higher than 2900 m (1487 toesas)’ [58]. In 1821 Humboldt stated the lower range was as low as 200 toesas, closer to Caldas' observations, although he reported one species at an altitude of 1500 m [59]. However, Humboldt never included Caldas’ observations in his essay [2]. Humboldt & Bonpland *Cinchona* historical sources (electronic supplementary material, appendix, dataset S4) were used to create a dataset. Nine locations of Humboldt & Bonpland were georeferenced; four locations were assigned to *C. officinalis* and five to *Cinchona*.

#### 2.1.3. Tafalla and Manzanilla

As for the Tafalla and Manzanilla collections in the Real Audiencia de Quito, localities were sourced from the original labels of the sheets in the MA Herbarium by Tafalla, Manzanilla and Pavón. The original descriptions made by Tafalla and Manzanilla in Ecuador [60] in a copy of the Nueva Quinología of José Pavon manuscript and its publication [62] are preserved at the Real Jardín Botánico, CSIC Archives [61]. Tafalla and Manzanilla's herbarium sheets are part of Ruiz and Pavón's materials in the archives [63]. Tafalla and Manzanilla's records of *Cinchona* are compiled as one collection made up of herbarium sheets, descriptions, wood and bark samples labelled with a collection number [51]. These materials are related to Pavon's Nueva Quinología work. We selected the latest taxonomic data in the herbarium sheets, separated into two groups: (i) *C. officinalis* and synonyms; (ii) other species of *Cinchona* under the tag Cinchona. We checked the localities in the herbarium labels with those in the original descriptions of Tafalla and Manzanilla. From this data we produced a dataset of *Cinchona* distributions with a total of 45 locations (electronic supplementary material, appendix, dataset S4b). Twenty-seven localities were assigned to *Cinchona* sp. and 18 to *C. officinalis*.

In summary, all three sources of information, Caldas; Humboldt and Bonpland; and Tafalla and Manzanilla, produced 84 data entries gathered from Colombia and Ecuador (electronic supplementary material, appendix dataset S4). Not all those entries contained detailed information on geographical location, hence latitude and longitude were not assigned to all cases. As a result, only 61 georeferenced locations were included (seven from Caldas, nine from Humboldt & Bonpland and 45 from Tafalla & Manzanilla) (electronic supplementary material, appendix dataset S4).

### 2.2. Contemporary records

**Figure 4.**
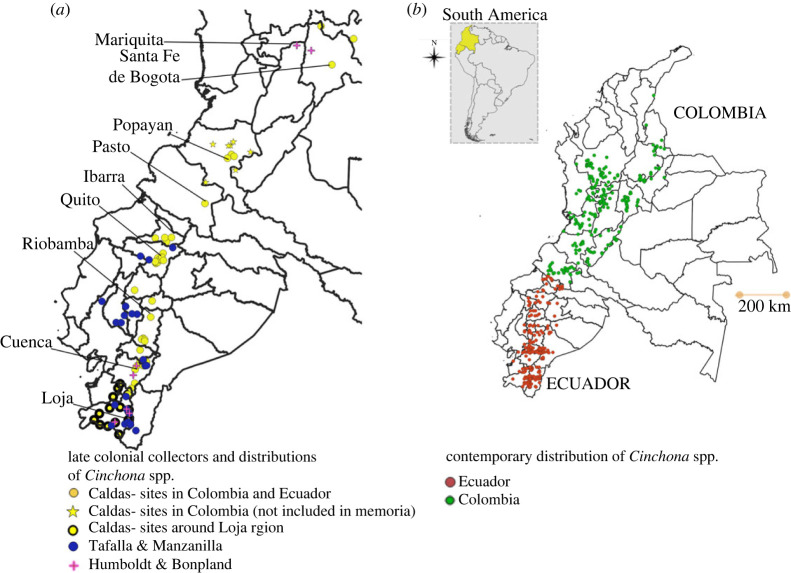
Distribution of *Cinchona* in Colombia and Ecuador. Late colonial collectors (*a*). Current distribution (*ca* 2000) (*b*).

The dataset of *Cinchona* and *C. officinalis* in Colombia compiled of data from the twentieth century referrred to as the ‘contemporary’ period, consisted of 668 entries (electronic supplementary material, appendix, dataset S3) [7]. Only records without known georeferenced issues were selected. This dataset was not used for any statistical or spatial analysis, rather it was used to illustrate the current distribution in Colombia as shown in figure 4*b*.

The contemporary dataset for Ecuador (electronic supplementary material, appendix, dataset S1) was developed from the geographical coordinates of 12 *Cinchona* species distributed across Ecuador. This is the most complete and accurate dataset of the genus because it comes from validated field sampling data of *Cinchona* conducted by Garmendia [6]. Each record in this dataset was digitized in a dynamic table of Excel and then mapped using QGIS Desktop v. 3.10.2. [64]. This dataset was used for the statistical analysis and consisted of 446 entries corresponding to herbarium sheets of the genus collected across Ecuador containing latitude, longitude and elevation for each entry (electronic supplementary material, appendix, dataset S1). No species or distributional data of *Cinchona* from commercial plantations were included in our study.

### 2.3. Statistical analysis

Two statistical analyses were conducted using Tool Graph Pad (https://www.graphpad.com). First, a linear model (LM) was used to establish the change in average elevation of *Cinchona* between historical periods in Ecuador and the relationship of elevation and latitude (electronic supplementary material, appendix, dataset S1). For the elevation and latitude analysis, the model included elevation as the response variable, period as categorical variables, and latitude as a covariable variable. In the second analysis, the difference between elevational ranges and average elevation between the periods was analysed using a Student's *t*-test for *Cinchona* distributions. The statistics used here were previously tested and validated in a similar study [3]. Both tests were applied firstly to Caldas's standalone dataset (electronic supplementary material, appendix, dataset S2b) and to the three historical collectors' combined datasets for data only in Ecuador (electronic supplementary material, appendix, dataset S6). Colombia's datasets were not analysed statistically because they did not have the same level of detail as the data collected in Ecuador (electronic supplementary material, appendix dataset S3). Finally, a student's *t*-test was applied to the mean elevation of a subset containing the *C. officinalis* records in the Loja region of Ecuador (a subset of electronic supplementary material, appendix, dataset S2a).

## 3. Results

### 3.1. Elevational limits

The upper and lower elevational limits of *Cinchona* reported by Caldas in Ecuador were 3164 and 290 metres above sea level (m.a.s.l.), respectively. The contemporary elevational limits were 3510 m.a.s.l. for the upper limit and 484 m.a.s.l. for the lower limit (electronic supplementary material, appendix, dataset S2). In Colombia, Caldas reported *Cinchona* in Popayan, Pasto and the surrounding areas of Santa Fe de Bogotá with an upper limit of 2700 m.a.s.l. The limits of *C. officinalis* reported by Caldas around the Loja region of Ecuador were 1310 and 2738 m.a.s.l., respectively. The upper and lower limits during the contemporary period in the same region were 1450 and 3330 m.a.s.l. (electronic supplementary material, appendix, dataset S2).

### 3.2. Distributional datasets

A total of 319 distributional data of *Cinchona* recorded by Caldas; Humboldt and Bonpland; and Tafalla and Manzanilla during the late colonial period in Ecuador and Colombia are presented in table 1 and figure 4*a*. Most of these records were collected by Caldas in Ecuador, where his most extensive and detailed collections were performed. A total of 1114 records were reported for the contemporary period (table 1 and figure 4*b*).

### 3.3. Changes in elevation and latitude

**Figure 5.**
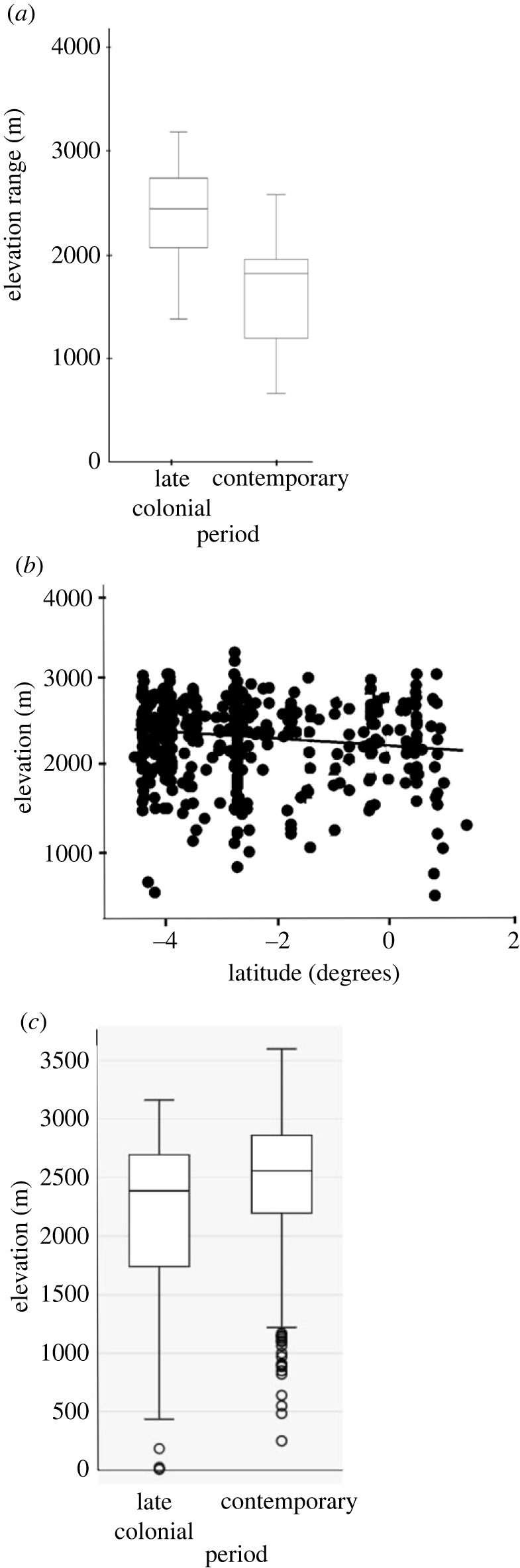
Changes in elevation and latitude of *Cinchona* collections in Ecuador in the late colonial and contemporary periods. (*a*) Box and whiskers plot of the elevational ranges (maximum–minimum elevations); *C. officinalis* elevation ranges reported by Caldas were significantly different between periods (Student's *t*-test, *t* = 4.44, d.f. = 62, *p* < 0.0001). (*b*) Linear model (adjusted *R*^2^ = 0.01968; *p* = 0.0018; *n* = 493) showing the relationship between elevation and latitude of *Cinchona* in Ecuador. (*c*) Box and whiskers plot of the average elevation changes of *Cinchona* reported by Caldas, Humboldt & Bonpland and Tafalla & Manzanilla. The changes were significantly different between periods (Student's *t*-test, *t* = 4.00, d.f. = 104.83; *p* < 0.0007123).

The comparison between the historical and the contemporary distributions of *Cinchona* revealed two findings. First, there has been a significant expansion of the genus and *C. officinalis* in elevational range and average elevation since the late colonial period in Ecuador. We found that the *C. officinalis* elevational range change reported by Caldas in the Loja region was significantly different between periods (Student's *t*-test, *t* = 2.62, d.f. = 47, *p* = 0.0117) with an overall range change of 792.5 m.a.s.l. (figure 5*a*). The average elevation of *Cinchona* reported by the three historical collectors (electronic supplementary material, appendix, dataset S6) was also significantly different between periods (figure 5*c*), with an average of 662.5 m.a.s.l. These values are similar to an expansion of 740.1 m.a.s.l. found in a study on the elevational range of eight crops recorded by Caldas in the same geographical region [3]. Second, the elevational distribution of the genus decreases when moving south (figure 5*b*).

## 4. Discussion

### 4.1. Distributional and elevational patterns

The changes in elevation and latitude apply mostly to regions between 1° N to 5° S in Colombia and Ecuador and do not apply to the full distributional range of the genus. We found a substantial expansion and significant distributional changes for both the *Cinchona* and *C. officinalis* in Ecuador; however, we could not prove the directionality of these changes. Based on our results (figure 5), it is inferred that a bidirectional shift in elevation, upward or downslope, could have occurred over time. We could argue that a downslope shift could be related to changes in dry forest areas where natural habitats have been heavily impacted during the last decades. In contrast, the potential upward shift could be attributed to the mountaintop effects caused by land-use changes, particularly due to agriculture and global warming in the northern Andes [65,66]. The elevational distribution of *Cinchona* was affected by latitude: as latitude goes south, the genus elevation increases. There is some evidence that changes in elevational distribution could be attributed to historical factors and ecological conditions favouring range restricted species distributed in high elevations towards the south of Ecuador.

### 4.2. Potential drivers of distributional changes

The large expansion of the average and elevational range of *Cinchona* and *C. officinalis* over 200 years suggests that species are responding to human impacts, including global warming. The majority of the distributional records of *Cinchona* were reported over 2000 m in elevation. Assuming that they have been modified as a result of climate effects or human impacts, we could argue that the genus *Cinchona* experienced an upslope shift over two centuries, in accordance with similar studies on the Andean region [65–67]. On the other hand, a major increase of the elevation range of *Cinchona* on the lower elevational limits, below 2000 m, suggests a downslope trend, which deviates from many studies on upper Andean vegetation. This could be also due to deforestation occurring in forests along the mid to low elevations where it is more populated. On the other hand, this trend could be related to the change known as range-shift gaps, associated with the fact that tropical lowland species can gain distribution range due to having a wider niche breadth than the top mountain species [67]. The combination of those trends explain the large overall expansion of the genus over two centuries.

The expansion of the agricultural frontier has caused large land-use changes in the Neotropics, particularly in the northern Andean forests [68]. Heavy exploitation of *Cinchona* bark has occurred since the seventeenth century, starting in the Loja region. Deforestion where *Cinchona* grow have been reported since the eighteenth century. In the nineteenth century, those pressures intensified and spread. This acted in synergy with other drivers of land-use change, such as the expansion of the agricultural frontier, at times associated with the introduction of grasses for pastures changing the mountain landscapes in Colombia and Ecuador [69–71].

Quina trees in middle elevations were heavily impacted by argicultural burning, which was common practice mentioned by Caldas. However, upper forests were still in good ecological conditions for *Cinchona*. This was confirmed by Caldas's collections found in higher elevations than the contemporary records. Due to agricultural expansion in the mountains of the Andes, habitats in upper elevations became more vulnerable once colonization progressed, threatening the remaining forest in the upper end of the ecological niche of the genus, as our results proved.

Increasing temperatures may trigger elevational changes in species distributions [65]. These may have caused a contraction of the current elevational range of the genus (figure 5), as we observed in Ecuador, resulting in an upward shift and eventually the disappearance of *Cinchona* habitat in the mountaintops [72]. Our evidence points to *Cinchona* contracting and shifting from the top of the Andes towards the middle elevations (figure 5). This could be related to deforestation of dry forests in the southern mountains of Ecuador in the province of Loja where certain species of *Cinchona* thrive. For instance, a study on the *Cinchona* species suggests that under a representative concentration pathway (RCP) scenarios of 2.6 and 4.5, *C. pubescens* climate suitability areas will increase by the year 2070, but its changes are expected to distribute in small and fragmented patches [8]. Fragmented forests of *Cinchona* in the lowlands of Ecuador have experienced reductions which could be related to a decrease of habitat. Garmendia mentioned in his study that in the Loja valleys *C. officinalis* forests are not present any more. This does not mean that the species have disappeared but shows that its habitat has reduced, diminishing the number of species.

Finally, ecological processes such as colonization and germination could play a big factor for promoting or restricting species range shifts [53]. Habitat in the upper limits of *Cinchona* distribution has declined in the last two centuries in Ecuador and Colombia due to agricultural expansion. Crops like potatoes are planted at high elevations closing the gap between residual fractions of forests and the farmers' land. Many species of *Cinchona* such as *C. pitayensis*, *C. macrocalyx, C rugosa* and *C. mutisii* are distributed in the higher elevations of the Andes in Ecuador. A reduction of habitat closer to the mountaintops due to global warming [73,74] will affect the capacity of species to colonize new habitats or migrate and could result in the disappearance of populations altogether. If the habitat is fragmented, these processes will probably cause direct negative effects on the species' capacity to disperse or to migrate to higher elevations. Having low dispersal, especially in species restricted to the Andean range, could lead to lower seed germination compromising the populations.

### 4.3. Uncertainties and biases of *Cinchona* historical collections

The full distributional range or taxonomic diversity of *Cinchona* was not covered in this study. Only 12 species of the 23 accepted taxa in the genus were analysed as part of our study. We used just half of the accepted species resulting in some distributional and taxonomic bias. Despite this, our results account for about half of the *Cinchona* distributional range and taxonomic diversity in Colombia and Ecuador and, more specifically, for Ecuador between 1° N and 5° S of latitude.

**Figure 6.**
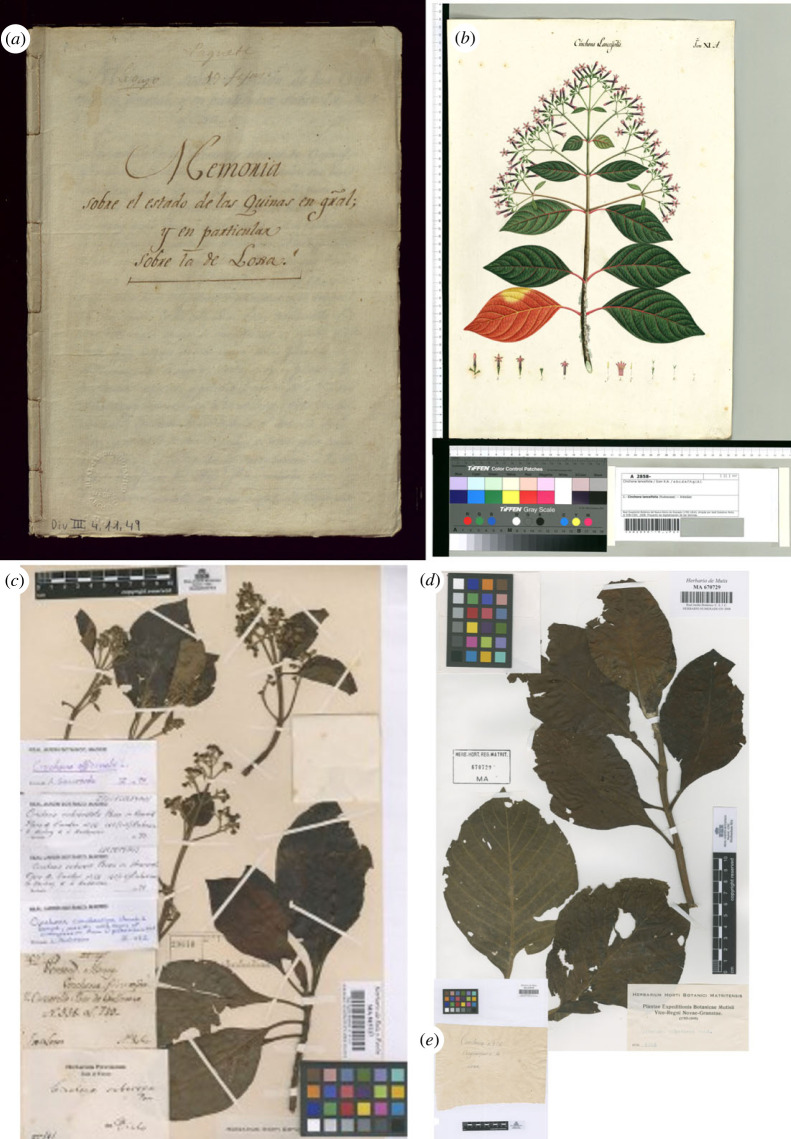
Examples of *Cinchona* botanical work conducted by Caldas (Royal Botanical Expedition to the New Kingdom of Granada) and J. J. Tafalla and J. A. Manzanilla (Royal Botanical Expedition to the Viceroyalty of Perú). (*a*) Front cover of the original *Memoria sobre el estado de las quinas en general y, en particular, sobre las de Loja*, written by Caldas, Quito, 16 March 1803. AJB, Div. III, 4, 11, 49; (*b*) *C. lanceifolia* drawn by an anonymous artist from the Royal Botanical Expedition to the New Kingdom of Granada based on Caldas's materials. Bogotá, 1805–1808. AJB, III, 2858; (*c*) specimen of *C. officinalis* collected by Tafalla and Manzanilla in Loja, 1805, identified by several different taxonomists. MA815727; (*d*) specimen of *C. pubescens*, collected by Caldas in Loja, 1801–1805. MA670729; (*e*) original label by Caldas: ‘Cinchona n. 124 Chahuarguera de Loxa’ documented in the herbarium sheet MA670729. Images courtesy of the Real Jardín Botánico, CSIC.

In terms of taxonomic accuracy, the genus *Cinchona* has received different revisions, some that were published [5] and others made only to herbarium specimens. Several specimens have been given different identifications at the species level, including by the same researcher in a short period of time. For instance, the herbarium collections shown in figure 6*c* have been identified by different authors as firstly *C. suberosa*, then *C. condaminea*, then again *C. suberosa* and finally *C. officinalis*. Figure 6*d,e* shows a specimen with its corresponding label as *C. pubescens* collected by Caldas. Although molecular identification techniques are gaining ground, for this research we relied on the latest identification available for each specimen of the genus *Cinchona*, and *C. officinalis*.

Another potential source of bias could have occurred when retrieving the historical data. We found that Caldas's maps and publications provided the most detailed information of all the collectors from the late colonial period. Despite an extensive and meticulous search of Caldas's maps, there was some uncertainty on whether the correct scientific name had been recorded and the lack of records. For instance, in the case of Colombia, only a few historical records of *Cinchona* were reported for the late colonial period. In contrast, the historical data for Ecuador is robust (table 1).

Distributional biases also may exist due to the limited area we studied. Venezuela, Peru and Bolivia are also part of the natural distributional range of *Cinchona*; however, collections in those countries are not well documented. Consequently, those countries were excluded from the study.

Most of the distributional data of *Cinchona* contained in historical archives lack detail. We used information such as names of towns or mountains that have not changed since the colonial period to identify the latitude and longitude of locations. This information can be reliably used to generate the geolocations in a digital format. In other cases, species locations can be identified through museums and herbaria records.

The colonial paths and modern road networks in the Andean region could have also caused bias. However, we did not notice linear shapes in the historical distributions and therefore spatial information provided by one collector could not be confirmed with another collector's records of the same area. Caldas's records covered the widest territories. Tafalla & Manzanilla on the other hand, sampled in clusters more often at lower elevations. Humboldt & Bonpland collected in specific localities such as Santa Fe de Bogota.

The upper and lower elevational limits of *Cinchona* could be another source of uncertainty because elevational limits have been constantly changing through time. However, we can argue that our results capture some degree of the distributional dynamics of the genus.

The interpretation of historical data to evaluate changes in the distribution of plants poses several challenges. The first one is to establish a coherent dialogue between history, biogeography and taxonomy. Understanding the potential and limits of registers from the past and the ways to match them with contemporary data is a challenge, as no methodology can be applied to all cases. Previous attempts to make such comparisons in natural and cultivated plants have been made [72], a work that was also not exempt from problems, as has been observed by other researchers [37], who support a ‘cautious approach needed to interpret historical data and to use them as a resource for documenting environmental changes’.

A different set of challenges was found for the contemporary dataset of *Cinchona*. There are not many records reported in Colombia, and so our focus was mostly limited to Ecuador. Garmendia's dataset of Ecuador provided better sampling accuracy and validated taxonomic data [6]. We also cannot with full certainty confirm species names because they are not well curated. The taxonomy and nomenclature of *Cinchona* are still in development, and therefore our study did not consider intra-specific information or hybrids. Furthermore, different taxonomic identifications in the *Cinchona* group added to the uncertainty. For this reason our main analysis was conducted at the genus level.

## 5. Conclusion

Using historical archives and botanical collections from the nineteenth and twentieth centuries provided sufficient evidence that a large expansion in the distribution of the genus *Cinchona* and *C. officinalis* along latitude and elevation has occurred in Ecuador. This demonstrates the contribution historical records can make to understanding the past and present distribution of important species.

## Data Availability

The data are provided in electronic supplementary material [75].
